# Selection of anesthetic agents for obstructive jaundice: An update for anesthesiologists

**DOI:** 10.1097/MD.0000000000043074

**Published:** 2025-06-27

**Authors:** Yi-Qing Li, Xuefei Zhou, Yunfei Cao, Yue Long

**Affiliations:** a Department of Anesthesiology, Ningbo Medical Center Lihuili Hospital, Ningbo University, Ningbo, China; b Department of Anesthesiology, Beilun District People’s Hospital of Ningbo, Ningbo, China; c Department of Anesthesiology, 921 Hospital, Joint Service Support Force of People’s Liberation Army, Changsha, China.

**Keywords:** anesthesiologists, anesthetic agents, obstructive jaundice, update

## Abstract

Obstructive jaundice is induced by complete or partial obstruction of the common bile duct, which causes bile to overflow from the digestive system into the circulatory system. Clinically, such patients often require surgical interventions to relieve obstructive jaundice, but the extensive and complex pathophysiological changes associated with obstructive jaundice not only lead to a higher incidence of perioperative morbidity and mortality, but also significantly affect the pharmacological properties of anesthetic agents, which poses a major challenge to anesthesia management. Therefore, this article reviews the research progress on the pharmacological properties of anesthesia in obstructive jaundice in the past 20 years, and focuses on the influence of obstructive jaundice on the pharmacodynamics and pharmacokinetics of inhaled anesthetics, intravenous anesthetics, opioid analgesics, and muscle relaxants commonly used in clinic. In fact, alterations in pharmacological properties of these anesthetic agents induced by obstructive jaundice vary widely. For example, patients with obstructive jaundice have increased sensitivities and decreased requirements to desflurane, etomidate, and rocuronium, while propofol and atracurium are almost not affected. In addition, the effects of these drugs on the functions of vital organs in jaundiced patients are also discussed in detail. Anesthesiologists should be aware of the importance of rational use of anesthetic agents in jaundiced patients during operation, and the selection of appropriate anesthetics on the basis of comprehensive evaluation and the precise adjustment of dosage based on strict intraoperative monitoring, are crucial to ensure the stability of anesthesia and reduce perioperative risks.

## 1. Introduction

Obstructive jaundice, also known as surgical jaundice, is a common pathological condition characterized by increased serum bilirubin levels due to partial or complete mechanical obstruction of intrahepatic or extrahepatic bile ducts.^[1,2]^ Choledocholithiasis is the primary cause of this obstruction, with other contributors including periampullary cancers, inflammation, trauma, bile duct stenosis, and even congenital biliary atresia.^[1,3]^ Patients with obstructive jaundice usually require surgical interventions to relieve the primary lesion and obstruction, and many of them have high preoperative bilirubin levels and often need to undergo endoscopic retrograde cholangiopancreatography or percutaneous transhepatic drainage first to reduce jaundice levels and minimize perioperative risks.^[4,5]^ However, even such preoperative intervention usually needs to be performed under anesthesia to enhance the patient’s cooperation and comfort, and to facilitate procedural manipulations.^[1,4]^ Therefore, anesthesiologists still have to face these complex and specific patients with severe jaundice.^[2,6]^ The diverse pathophysiological derangements and systemic toxic effects related to obstructive jaundice make the perioperative management markedly distinct from other patient populations, which may easily be associate with high perioperative morbidity and mortality, and pose considerable challenges to anesthesiologists, surgeons, and intensive care teams.^[1,2]^

Fortunately, over the past 2 decades, ongoing exploration, predominantly from Chinese scholars, has systematically elucidated the anesthetic specificity of obstructive jaundice, including alterations in anesthetic sensitivity, reductions in pain threshold, changes in pharmacokinetics, and the effects of anesthetics on cardiopulmonary and renal function in jaundiced patients. These achievements and updated insights are of great importance for clinical practice, and anesthesiologists can accordingly select optimal perioperative anesthesia management for jaundiced patients, so as to minimize perioperative risks and contribute a favorable outcome.

## 2. Selection of inhaled anesthetics for obstructive jaundice

Neurological symptoms, encompassing impairments in mental status, intellectual functioning, alertness, and motor function, are frequently observed in patients with obstructive jaundice, and may further progress to “hepatic encephalopathy” or coma as jaundice persists or worsens.^[7]^ Animal studies have also confirmed that cholestatic jaundice was linked to spatial deficits in rats with bile duct ligated for 3 weeks, and these cognitive deficits could be reversed by biliary drainage.^[8]^ Obviously, these pathological states of brain function induced by obstructive jaundice may also affect the sensitivity to certain anesthetics. Previous studies demonstrated a significant decrease in the minimum alveolar concentration for awakening (MAC_awake_) of desflurane in patients with obstructive jaundice as compared to non-jaundiced controls (1.78 ± 0.19% vs 2.17 ± 0.25%), with a highly inverse relation between MAC_awake_ of desflurane and serum total bilirubin concentration.^[9]^ Similar increased sensitivity to isoflurane and prolonged recovery time under sevoflurane anesthesia were also observed in patients with obstructive jaundice.^[10]^ Moreover, due to the increased sensitivity to anesthetic agents, patients with obstructive jaundice are prone to hypotension and bradycardia during the induction and maintenance of anesthesia. Therefore, a relative reduction in the dose of inhaled anesthetics is recommended for patients with obstructive jaundice, and intraoperative monitoring should be emphasized, especially for patients using a single inhaled anesthetics.

It is worth mentioning that there may also be central regional differences in the effect of obstructive jaundice on the sensitivity to inhaled anesthetics. Chen et al observed a reduction in MAC_awake_ of sevoflurane in infants scheduled for abdominal surgery with obstructive jaundice compared to nonjaundiced controls (1.00 ± 0.15% vs 1.40 ± 0.21%), and also a negative correlation between serum total bilirubin levels and the probability of awakening (OR = 0.984). Interestingly, no significant difference was noted in the MAC for endotracheal intubation (MAC_EI_) of sevoflurane between jaundiced and nonjaundiced infants.^[11]^ This discrepancy may be attributed to the distinct central target regions and mechanisms involved in MAC_EI_ and MAC_awake_ of isoflurane. As it is known that the target areas of anesthesia-induced hypnosis and amnesia are mainly located in the cerebral cortex, the enhanced hypnotic effect of isoflurane is more likely to be secondary to the altered corticocerebral functional state caused by obstructive jaundice, while MAC_EI_, which represents the antinociceptive reflex of anesthetics, is mainly located in the subcortical center. However, it is unclear whether obstructive jaundice has distinct effects on cortical and subcortical centers.

It is worth noting that the central pathological changes caused by obstructive jaundice may become irreversible as jaundice persists. Then, even if the duct obstruction is relieved or hyperbilirubin levels are lowered, the change in sensitivity to inhaled anesthetics may also be difficult to recover, which needs the attention of anesthesiologists, but no definite study has been reported so far.^[7]^ In addition, obstructive jaundice can often be accompanied by liver function impairment, so the selection of drugs with hepatorenal toxicity such as halothane should be avoided. Other commonly used inhaled anesthetics, such as enflurane, isoflurane, etc, have no obvious adverse effects on liver function, and there is no special contraindication for patients with hepatobiliary diseases such as obstructive jaundice. The study by Arenas YM et al also confirmed that sevoflurane used in hepatectomy for patients with cirrhosis did not cause damage to renal function.^[12]^

Although studies have shown that bile acids and their mediated signaling may play a role in inducing blood–brain barrier permeability,^[13]^ neuroinflammation,^[14]^ and exacerbating neurological decline,^[15]^ the exact cause or underlying mechanism of increased sensitivity to inhaled anesthetics in jaundiced patients remains unknown. As inhaled anesthetics such as isoflurane and desflurane have low blood solubility and little metabolism in vivo, and are predominantly eliminated by the lung rather than the liver and kidneys, their pharmacokinetic characteristics are unlikely to significantly differ in jaundiced patients, even with pathophysiological changes such as impaired liver function and extracellular fluid depletion.^[9,10,16]^ Thus, altered neurotransmission and neurotoxicity in the brain due to obstructive jaundice may play a role in the altered sensitivities of inhaled anesthetics. Studies in animal models have confirmed that obstructive jaundice can produce a wide range of neurotoxicity to brain tissue, naturally involving central action areas or targets of inhaled anesthetics. In a well-characterized canine model of cholestasis secondary to bile duct resection, histologic evidence of nerve damage, such as simple atrophy and pyknosis of nerve cells, neuronophagia, and ghost cells, was observed in the brain, and neurologic damage may extend from the basal ganglia, putamen and red nucleus to the thalamus, cerebral cortex and substantia nigra, with prolonged obstructive jaundice.^[17]^ Similar ultrastructural changes were observed in Wistar albino rats after bile duct ligation (BDL), including degenerated glial cells, apoptotic electron-dense neurons, and blood–brain barrier deterioration in the cerebral cortex, and the duration of obstructive jaundice correlated with the extent of neurologic damage.^[7]^ It was also confirmed that the impairment of brain functions such as memory and motor coordination in cholestatic rats was associated with alteration of neuroinflammation and GABAergic neurotransmission in the cerebellum.^[12]^ In fact, almost all classical neurotransmitter systems, including opioidergic, dopaminergic, cholinergic, GABAergic, adrenergic, serotonergic, and glutamatergic systems, have been found to be altered in both jaundiced patients or cholestatic animal models.^[18]^ For instance, defective serotoninergic neurotransmission, which was considered to be sensitive to inhaled anesthetics, may partly explain the reduced needs for anesthetic requirement in patients with obstructive jaundice. As midbrain somatodendritic 5-HT_1A_ autoreceptors, which play a central inhibitory role in the regulation of serotonergic neurotransmission, appears to be altered in rats with experimental cholestatic liver disease, as shown by enhanced 5-HT1A autoreceptor-mediated physiological responsiveness (hypothermia and bulimia) in the setting of increased midbrain 5-HT_1A_ receptor number but not affinity.^[19]^ Also, in cholestatic male Wistar rats induced by BDL, a decrease in memory retrieval occurred 11 days after BDL, and the possible involvement of dorsal hippocampal glutamatergic systems was conformed upon cholestasis-induced amnesia.^[7]^

In summary, the toxic effects of jaundice on the central nervous system, including histologic evidence of neurologic damage and altered neurotransmission in the brain, which are considered to be critical targets for inhaled anesthetics, may lead to increased sensitivity to anesthetics and reduce anesthetic requirements. However, it should be pointed out that the impact of obstructive jaundice on the central nervous system is different from that of bilirubin encephalopathy,^[2,15,16]^ the former is mainly caused by elevated bound bilirubin, while the latter is unbound bilirubin.

## 3. Selection of intravenous anesthetics for obstructive jaundice

Due to concerns about environmental pollution of inhaled anesthetics, intravenous anesthetics are the most commonly used in clinical practice. Therefore, for patients with obstructive jaundice, whether it is procedural sedation outside the operating room or general anesthesia inside the operating room, the choice of intravenous anesthetics is particularly important. Though obstructive jaundice can alter the sensitivities of most anesthetic agents, propofol appears to be an exception. Song JC et al adopted bispectral index and mean arterial pressure as indices of the anesthetic potency of propofol, revealing that obstructive jaundice within a serum total bilirubin ranging from 7.8 to 362.7 µmol/L had no discernible impact on propofol pharmacodynamics.^[20]^ In contrast, patients with obstructive jaundice required a lower amount of etomidate, another intravenous anesthetics, to achieve a predefined level of anesthesia compared to non-jaundiced controls, that is, patients with obstructive jaundice have increased sensitivity to etomidate. Moreover, a significant negative correlation was found between serum total bilirubin levels and etomidate requirements.^[21]^ The differential sensitivity of jaundiced patients to etomidate and propofol can be attributed, in part, to the distinct central targets of these 2 drugs,^[22,23]^ as studies have shown that propofol exerted anesthetic potency not only by binding to the β subunit of the postsynaptic GABA_A_ receptor, but also acted on other receptors (such as glycine, M_1_ muscarinic acid and nicotinic acid receptors),^[22]^ while etomidate is a pure hypnotic GABA agonist, and appears to produce hypnosis, amnesia, and inhibition of nociceptive responses almost exclusively via actions at GABA_A_ receptor.^[23]^ Additionally, animal studies have confirmed that bilirubin potentiated GABA/glycanergic inhibitory synaptic transmission in the lateral superior olivary nucleus neurons of rats, which may contribute to the increased sensitivity to etomidate induced by obstructive jaundice.^[24]^ Despite the potential impact of obstructive jaundice on liver function and drug-metabolizing enzymes,^[1]^ it is noteworthy that obstructive jaundice seemingly has no effect on the pharmacokinetics of either propofol or etomidate.^[25,26]^ A comparative study of patients with or without obstructive jaundice showed similarities in several pharmacokinetic parameters and halftime of the 3 phases [T(1/2) (alpha), T(1/2) (beta), T(1/2) (gamma)], suggesting that obstructive jaundice does not influence the pharmacokinetics of propofol.^[25]^ Moreover, moderate liver dysfunction does not appear to affect propofol clearance due to its existence of an extrahepatic metabolic pathway.^[27,28]^ Further studies affirmed that the pharmacokinetics of etomidate remained unchanged in the presence of obstructive jaundice, and a 3-compartment open model best described the concentration profile of etomidate after bolus infusion for anesthesia induction.^[26]^ However, the plasma clearance and distribution volume of dexmedetomidine, predominantly metabolized and excreted by the liver, were decreased by 33.3% and 29.2%, respectively, in patients with obstructive jaundice as compared to non-jaundiced patients.^[26]^ The changes of liver blood flow and the damage of drug metabolism enzyme system caused by obstructive jaundice may contribute to the decrease of plasma clearance and distribution volume of dexmedetomidine. Therefore, the use of dexmedetomidine in patients with obstructive jaundice also requires dose adjustment.

Both clinical and animal studies have demonstrated that obstructive jaundice can impair the liver’s drug-metabolizing enzyme system. In rat models, the content of total cytochrome P450 significantly decreased after bile duct ligation and recovered after bile duct drainage.^[29]^ However, the components of cytochrome P450 family are extremely complex, and the impact on different components can vary significantly in response to obstructive jaundice. For instance, CYP3A4 isoform is an essential member of the liver cytochrome P450 enzyme family, which is mainly responsible for metabolizing a variety of intravenous anesthetics and sedative agents (including diazepam, midazolam, fentanyl, lidocaine, etc).^[30]^ The isomeric activity of CYP3A4 is significantly decreased in patients with cirrhosis, but obstructive jaundice alone does not affect the activity of CYP3A4, and the significant changes in the activities of these metabolic enzymes may primarily occur in the biliary cirrhosis or liver failure associated with obstructive jaundice.^[31]^ However, the effects of obstructive jaundice on the pharmacokinetics and metabolites of many intravenous anesthetics metabolized by liver, such as benzodiazepines, dexmedetomidine, ketamine, etc, have not been reported so far.

Although propofol has outstanding clinical advantages and its pharmacokinetics and pharmacodynamics are not affected by obstructive jaundice, it is still controversial whether propofol is suitable for patients with obstructive jaundice due to its obvious cardiovascular inhibition, and jaundiced patients are prone to hypotension and bradycardia when induced and maintained with propofol.^[6]^ Animal studies indicated that propofol depressed cardiac parameters similarly in bile duct-ligated and sham-operated rats at low and intermediate doses, and only at a high dose, might propofol cause severe cardiac depression in jaundiced rats.^[32]^ Interestingly, propofol itself also has a certain protective effect on cardiovascular function.^[25]^ Clinical observations have shown that, compared with isoflurane or low-dose propofol, intravenous administration of high-dose propofol during cardiopulmonary bypass could reduce postoperative myocardial cell damage,^[33]^ but its specific mechanism remained unclear. Overall, the current consensus is that low and intermediate doses of propofol are safe for jaundiced patients.^[34]^

For patients with obstructive jaundice, etomidate is another intravenous anesthetics that can be considered. However, the pharmacodynamics of etomidate may be affected by obstructive jaundice, so its clinical dose should be adjusted or reduced accordingly in jaundiced patients. Of course, etomidate also has its advantages, prominently manifested in its less impact on hemodynamics, for instance, during endoscopic retrograde cholangiopancreatography anesthesia, the hemodynamics of etomidate are more stable than those of propofol, and the choice of etomidate can help avoid severe hypotension.^[35]^

In addition, contrary to the hepatotoxicity associated with some inhaled anesthetics, many intravenous anesthetics offer potential benefits for obstructive jaundice. As in cholestatic rat models, anesthesia with ketamine and propofol induced minimal renal tissue oxidative stress, and the protective effect and importance of these intravenous anesthetics on free radical damage of renal tissue in jaundiced patients during perioperative period should also be considered, which may help prevent the occurrence of postoperative acute renal failure.^[36]^ Additionally, It has also been reported that obstructive jaundice may severely affect the biochemical and immune function, while dexmedetomidine can reduce the concentration of inflammatory mediators IL-6 and TNF-α through PI3K/Akt/HIF-1α signaling pathway, and alleviate lung injury in jaundiced rats.^[37]^

## 4. Selection of analgesic for obstructive jaundice

It has long been found that obstructive jaundice is often accompanied by an increase in the level of plasma endogenous opioids. In cholestatic rats with bile duct resection, total plasma opioid activity was 3-fold greater than that in sham-operated controls, and plasma methionine-enkephalin levels were more than 6-fold greater than in sham-operated controls and more than 17-fold greater than in unoperated controls, indicating the profound impact of cholestasis on endogenous opioid levels.^[38]^ Similarly, Tao K et al confirmed that the plasma level of β-endorphin was increased in jaundiced patients as compared with non-jaundiced group (286.6 ± 14.5 vs 193.9 ± 9.6 pg/mL), though no significant differences were observed in cerebrospinal fluid and liver tissue concentrations between the 2 groups.^[39]^ Other studies showed that elevated plasma endogenous opioids may contribute to the pathophysiology of cholestasis, such as cardiovascular, liver, and renal function impairment, as well as pruritus and antinociception.^[40]^

The elevated endogenous opioid levels accompanied with obstructive jaundice and the resulting antinociceptive effects (analgesia), also altered the sensitivity and need for opioid analgesics, and had a greater impact on perioperative analgesia. The study by Yang LQ et al demonstrated decreased intraoperative requirements of isoflurane and remifentanil in jaundiced patients.^[6]^ And patients with obstructive jaundice showed a higher pain threshold for electrical stimulation(1.7 ± 0.3 vs 1.1 ± 0.1 mA), whereas 48-h postoperative morphine consumption was approximately 50% higher in none-jaundiced patients than that in jaundiced patients.^[40]^ All these studies suggested that perioperative opioid analgesics should be appropriately reduced in patients with obstructive jaundice. However, plasma β-endorphin levels did not correlate with electrical pain thresholds or 48-hour morphine consumption, suggesting the involvement of other endogenous opioids, such as met-enkephalin, in the increased pain threshold associated with obstructive jaundice.^[40]^ Research by Ahmadi S et al further confirmed that cholestatic rats showed significant antinociception 2 weeks after bile duct ligation which could be prevented by naloxone, and the expression of µ-opioid receptor-1 gene in the central hypothalamus, prefrontal cortex and hippocampus was significantly decreased, while no significant change was observed in the striatum.^[41]^ However, in general, central opioidergic tone appears increased in cholestasis syndrome, possibly due to the increased availability of endogenous opioid agonists at opioid receptors in the brain, as the opioidergic system has been shown to play a role in some cholestatic-induced behaviors, such as the impairment of learning and memory, anxiolytic-like behaviors, alterations in sleep pattern, and tremors.^[42,43]^ So it is speculated that changes in endogenous opioid levels and their effects on opioid gene expression in certain brain regions may underlie changes in pain perception and other possible pathological changes (such as pruritus) caused by obstructive jaundice.

However, Nelson et al reported that cholestasis-associated antinociception may involve the local action of endogenous opioids (e.g., enkephalins synthesized in the skin) at sensory nerve endings.^[44]^ In addition, unlike isoflurane, altered sensitivity to remifentanil in obstructive jaundice may not primarily occur through central mechanisms, as cholestatic mice demonstrated antinociception to thermal and mechanical stimuli, which can be reversible by naloxone or naloxone methiodide, a naloxone derivative which does not cross the blood-brain-barrier, indicating an opioid antinociceptive effect mediated outside of the central nervous system.^[45]^

Elevated β-endorphin levels in obstructive jaundice may have a role other than antinociception, such as being associated with cardiovascular disease, as β-endorphin can inhibit myocardial contractiles and promote capillary dilatation in a variety of pathologic processes.^[46]^ However, fatigue and pruritus accompanying cholestasis can result from a central mechanism involving increased opioidergic neurotransmission.^[47]^

Although all opioid analgesics can cause biliary sphincter spasm and an increase in bile duct pressure, the adequate postoperative analgesia should be provided after the surgery. Anesthesiologists and surgeons could use a small dose of opioid analgesics, while the use of non-steroidal anti-inflammatory drugs should be avoided.

## 5. Selection of muscle relaxants for obstructive jaundice

For the choice of muscle relaxant in patients with obstructive jaundice, succinylcholine, a depolarizing muscle relaxant, is not recommended due to its potential to excite the vagus nerve, leading to bradycardia or ventricular arrhythmia. In addition, succinylcholine is hydrolyzed by butylcholinesterase (pseudocholinesterase) in blood and liver, and its metabolism and excretion are slowed down in the case of obstructive jaundice with impaired liver function, so more attention should be paid to the reduction of use, and it is advisable to prefer short-acting non-depolarizing muscle relaxants.^[48,49]^

At present, non-depolarizing muscle relaxants are widely used in clinic, among which atracurium and cisatracurium are the preferred muscle relaxants for jaundiced patients as their metabolism is independent of liver and kidney functions. Approximately 80% of these drugs are degraded via Hoffmann elimination, a very small number is broken down through acetate metabolism, and the remaining 15% is excreted via the kidney in a prototype form. There is no significant difference in the efficacy of these 2 muscle relaxants between genders and age groups, and repeated use does not lead to drug accumulation.^[50]^ However, the intensity of cisatracurium is 4 times that of atracurium, whereas its metabolite laudanosine is only one-third at an clinical equivalent dose, with no dose-dependent effect of histamine release and cardiovascular adverse effect.^[50,51]^ Thus, cisatracurium is advantageous for use in patients with obstructive jaundice.

Other types of non-depolarizing muscle relaxants, such as rocuronium bromide, are also widely used in clinical anesthesia for its short onset time and intermediate duration of action. However, rocuronium is primarily metabolized by the liver, eliminated by the biliary tract, and 9% is excreted by the kidney in its prototype form,^[52]^ so its use in patients with liver and bile diseases will be mixed with more complicated interfering factors. The study by Wang ZM et al showed that neuromuscular blockade of rocuronium was prolonged in patients with obstructive jaundice, while its onset time was not affected. Though the exact reason for the prolonged action time of rocuronium induced by obstructive jaundice is not completely clear, the impedance of rocuronium excretion may be the primary reason, probably due to bile duct obstruction and increased plasma unbound rocuronium competing with free bilirubin for albumin binding.^[53]^

Considering that the drug-metabolizing enzyme system may be impaired in jaundiced patients with plasma clearance of rocuronium bromide significantly reduced, rocuronium should be used cautiously during perioperative period, including an appropriate extension of the interval for drug supplementation, strengthening intraoperative muscle relaxant monitoring, and recommending the use of muscle relaxant antagonists to reduce the incidence of pulmonary complications and postoperative residual neuromuscular block.^[54,55]^ Among the commonly used muscle relaxant antagonists, Sugammadex^[51,54]^ or anticholinesterase^[55]^ are both recommended. However it is important to emphasize that neostigmine is a cholinergic drug, and therefore should be used with caution in jaundiced patients to prevent severe arrhythmias or cardiac arrest. As for sugammadex, it is more effective than neostigmine at reducing residual neuromuscular weakness, however whether obstructive iaundice affects its antagonistic potency or pharmacodynamic characteristics has not been reported so far.

## 6. Summary

In conclusion, the extensive and complex pathophysiological changes in patients with obstructive jaundice pose a major challenge for perioperative management. For anesthesiologists, the selection of appropriate anesthetics based on comprehensive properative assessment and the fine adjustment of anesthetic dosage according to vigilant intraoperative monitoring, are essential to ensure the stability of anesthesia and minimize perioperative risks. Of course, this article only reviews the selection of anesthetic agents for obstructive jaundice, but in fact, the pathophysiological changes of obstructive jaundice associated with perioperative anesthesia management involve almost all vital organs, including alterations in blood biochemistry and metabolism, coagulation, infection, liver injury, renal dysfunction, cardiovascular instability, malnutrition, stress ulcer, bacterial translocation, immunosuppression and other potential complications, which may increase the mortality and morbidity during perioperative period. Furthermore, variations in the severity or duration of jaundice can also lead to marked changes in the pharmacological properties of many anesthetic agents affected by obstructive jaundice. Therefore, a multidisciplinary approach is imperative for the comprehensive perioperative care of such patients, ensuring a collaborative effort to cope with the diverse challenges posed by obstructive jaundice at different stages and severity.

## Author contributions

**Conceptualization:** Yunfei Cao.

**Project administration:** Yunfei Cao, Yue Long.

**Supervision:** Yunfei Cao.

**Writing – original draft:** Yi-Qing Li, Xuefei Zhou.

**Writing – review & editing:** Yunfei Cao, Yue Long.
